# Lung tumors and non-malignant airways from patients with chronic lung inflammatory disease bear distinct genetic and epigenetic disruptions corresponding to metabolic processes involved in senescence and methylation

**DOI:** 10.1186/2049-3002-2-S1-P82

**Published:** 2014-05-28

**Authors:** Emily Vucic, Kelsie Thu, Victor Martinez, Chiara Pastrello, John Yee, Igor Jurisica, Stephen Lam, Wan Lam

**Affiliations:** 1 Department of Integrative Oncology, British Columbia Cancer Research Centre, Vancouver, BC, Canada; 2 Princess Margaret Cancer Centre, University Health Network, Toronto, ON, Canada; 3 Department of Surgery, Vancouver General Hospital, Vancouver, BC, Canada; 4 Departments of Computer Science and Medical Biophysics, University of Toronto, Toronto, ON, Canada

## Background

Chronic obstructive pulmonary disease (COPD) is an inflammatory lung disease associated with a 10-fold increased risk of lung cancer (LC), independent of smoking-status. Together these diseases contribute tremendously to mortality worldwide. Based on the notion that chronic inflammation forms a specific cancer-promoting environment; we hypothesized that COPD and non-COPD related lung tumors are molecularly distinct, and that these differences may yield promising diagnostic and therapeutic targets. Since genes that sustain high-level DNA and RNA alterations are likely selectively altered in tumor systems, we applied an integrative multi-omics analysis to lung tumors from COPD and non-COPD patients. To assess the potential clinical application of these findings, we examined the methylation status of COPD+LC specific genes in non-malignant airways from COPD patients w/LC.

## Materials and methods

Multi-omics profiling (copy number, DNA methylation and gene-expression) was performed on 76 tumor and non-malignant lung tissues using genome-wide array platforms. To identify genes/pathways which sustain high DNA and RNA level changes in tumors, we developed an algorithm to generate “Integrated Scores” for each gene based on magnitude of concomitant DNA and mRNA alterations. Integrated Scores were applied to all downstream analyses. COPD+LC specific genes were further assessed at the level of DNA methylation in airways from 25 COPD patients w/ and w/out LC.

## Results

Gene set and pathway enrichment analyses of genes frequently and differentially disrupted at both DNA and RNA levels in COPD tumors revealed enrichment of transcription factor gene sets involved in inflammation-related cancer, oxidative stress and smoking response; the most significant being *PITX2 -*a putative methylation biomarker in LC, previously associated with senescence gene networks in COPD.

Pathways specifically disrupted in COPD tumors included those involved in inflammation (atherosclerosis, fibrosis and IL-17 signalling), DNA damage (14-3-3σ signalling) and synthesis of the methyl-donor, S-Adenosyl-methionine (SAM) - a critical cofactor involved in DNA and histone methylation. Five COPD+LC specific genes were also altered at the DNA methylation level in non-cancerous airways of COPD patients w/LC, including putative cancer epigenetic diagnostic biomarkers (*CCNDBP1*, *HPGD*), genes associated with oxidative response and COPD progression (*PPARGC1A*) and biosynthesis of SAM (*MAT2B*).

## Conclusions

Genetic and epigenetic disruptions in lung tumors and small airways of patients with COPD and LC are distinct from those in corresponding tissues from patients w/COPD or LC alone. Genes altered in airways and involved in lung tumorigenesis in COPD patients may serve as clinically relevant markers for predicting LC or as targets for chemoprevention therapies.

